# Stem/progenitor cell-based therapy for Duchenne muscular dystrophy

**DOI:** 10.3389/fcell.2025.1640275

**Published:** 2025-09-03

**Authors:** Tsukasa Tominari, Chaitra Sathyaprakash, Yoshitsugu Aoki

**Affiliations:** Department of Molecular Therapy, National Institute of Neuroscience, National Center of Neurology and Psychiatry, Tokyo, Japan

**Keywords:** duchenne muscular dystrophy (DMD), dystrophin, stem cell therapies, skeletal muscle, cardiac muscle

## Abstract

Duchenne muscular dystrophy is a genetic disease where loss of sarcolemma-associated protein, dystrophin, leads to progressive muscle wasting, and eventual loss of life from complications linked to cardiac deficits. Currently, numerous molecular therapies to restore dystrophin have entered clinical trials. However, the therapeutic benefits of these strategies in promoting tissue regeneration and reducing fibrosis remain limited. Stem/progenitor cell-based therapy in DMD patients is a promising strategy to promote muscle regeneration, though the conditions of transplantation and pre-treatments of numerous cell types are still being optimized. Several cell types with different properties and origins, such as myogenic stem/progenitor cells, mesenchymal stem cells (MSCs), and induced pluripotent stem cells (iPSCs), have been studied for treating DMD. Myogenic stem/progenitor cells derived from healthy donors are expected to restore the number of myofibers as well as dystrophin expression in DMD muscles. MSCs derived from various tissues, including umbilical cord, have immunosuppressive properties and are expected to ameliorate DMD phenotypes in combination with other gene therapies. In this review, we will summarize the challenges that must be overcome to allow for successful DMD muscle tissue regeneration and review the latest findings in stem/progenitor cell-based DMD therapy. We will focus on the pre-conditioning of cells for replacement therapies and treatment of the disease niche to improve muscle fiber integration.

## 1 Introduction

**FIGURE 1:**
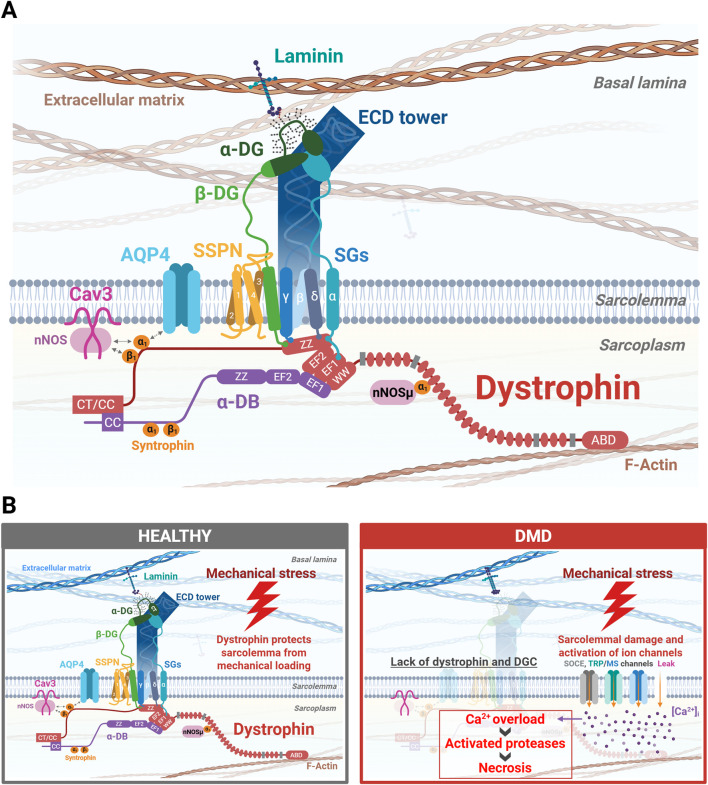
The role of the dystrophin-glycoprotein complex (DGC) in skeletal muscle. **(A)** The most recent structural model of the DGC is illustrated. Laminin, an extracellular matrix (ECM) protein, binds to the glycan of α-dystroglycan (α-DG) and anchors the DGC to the ECM. The β-dystroglycan (β-DG) interacts with extracellular α-DG and with dystrophin in the intracellular region. The extracellular regions of three sarcoglycans (SGs), including β-SG, γ-SG, and δ-SG, form a tower-like extracellular domain (ECD tower), which is thought to support the interaction between laminin and α-DG. The extracellular domain of α-SG attaches to the ECD tower. Sarcospan (SSPN) interacts with β-DG and SGs to stabilize the DGC at sarcolemma. The N-terminal of dystrophin (containing the actin-binding domain [ABD]) binds to F-actin of the cytoskeleton. The C-terminal (CT) of dystrophin binds to β-DG and α-dystrobrevin (α-DB) through WW-EF1/2 domains and the coiled-coil (CC) domain. Syntrophins, α1-syntrophin and β1-syntrophin, act as adaptor proteins for other proteins, such as neuronal nitric oxide synthase (nNOS) and aquaporin-4 (AQP4). AQP4 is a water channel mediating water transport. Caveolin-3 (Cav3) is a major component of caveolae, which acts as a scaffold to regulate various signaling pathways, including calcium signaling. At scale, the ECD tower is approximately 13.5 nm from base to head, not including the transmembrane region, and the dystrophin rod domains (24 rod domains) are around 120 nm. **(B)** Normal sarcolemma is stabilized by the DGC. Loss of dystrophin results in the breakdown of the DGC except for Cav3, causing sarcolemma instability and the activation of several calcium entry pathways, including the activation of ion channels such as store-operated calcium entry (SOCE), transient receptor potential (TRP) channels, and mechanosensitive (MS) channels. Elevated intracellular calcium ([Ca^2+^]_i_) levels trigger numerous cellular deficits in both early myoblasts and maturing myotubes, exacerbating the disease and compromising normal muscle regeneration. The figure was created with BioRender.com.

**FIGURE 2:**
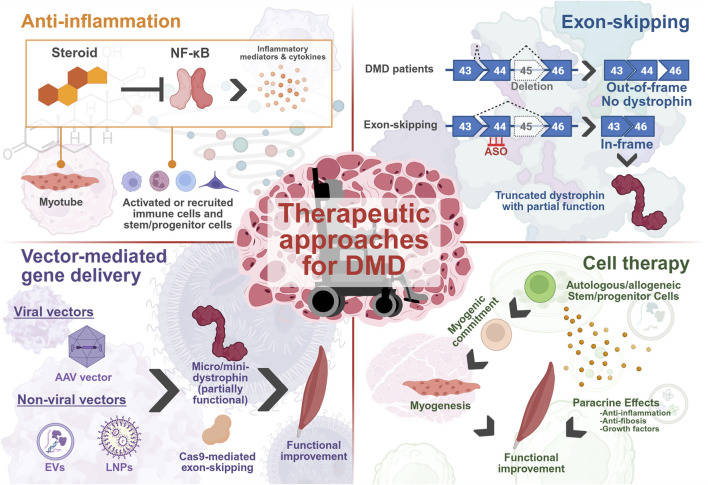
Current and developing treatments for DMD are targeted through several strategies, aiming to ameliorate the various disease phenotypes that manifest. These include reducing damage through inflammatory pathways via steroid-mediated inhibition of NF-κB, generating a partially functional, truncated dystrophin protein through exon-skipping or delivery of micro/mini-dystrophin mRNAs to improve muscle function by attempting to restore the DAPC and aiming to regenerate the depleted MuSC pool and muscle function by delivering healthy myogenic lineage cells to trigger local tissue repair. The figure was created with BioRender.com.

Duchenne muscular dystrophy (DMD) is an X-linked hereditary neuromuscular disorder caused by mutations in the *DMD* gene encoding dystrophin protein (Hoffman et al., 1987). DMD is characterized by severe and progressive muscle weakness, with an estimated incidence of approximately one in 5,000 male infant births. Dystrophin functions as an anchor between the basal lamina and the actin cytoskeleton through the formation of a dystrophin-glycoprotein complex (DGC) localized in the sarcolemma (Figure 1A) (Liu S. et al., 2024; Ozawa, 2010; Tsoumpra et al., 2019; Wan et al., 2024). Mutations in the *DMD* gene causing the loss of dystrophin in DMD patients lead to instability of the DGC. The latter becomes unable to support the sarcolemma against mechanical stress, resulting in abnormal calcium influx and muscle cell necrosis (Figure 1B). There is currently no cure for DMD. Instead, management of symptoms is prioritized through the administration of the FDA-approved steroids, prednisolone, deflazacort and vamorolone to delay disease progression, and significant support in daily life to reduce stress on skeletal muscle (Figure 2) (Jeon et al., 2025). Exon-skipping antisense oligonucleotide (ASO) therapies, which restore a partially functional, truncated dystrophin protein, have received approval for DMD therapy in Japan and the United States. These include eteplirsen for exon 51 skipping, golodirsen for exon 53 skipping, viltolarsen for exon 53 skipping, and casimersen for exon 45 skipping, and other exon skipping drugs that are currently in clinical trials (Figure 2) (Tominari and Aoki, 2023). Such treatments currently have limited restoration of dystrophin protein expression at approximately 20% of healthy individuals, as evaluated by Western blotting (Komaki et al., 2024). Consequently, the propensity to heal patient muscle tissue, which exhibits reduced muscle regeneration via endogenous injury response mechanisms and significant fibrosis, is still limited (Kemaladewi et al., 2014; Mogharehabed and Czubryt, 2023). This is caused by loss of dystrophin expression in premature myoblasts, which causes aberrant differentiation into mature myotubes and continuous cycles of pro-inflammatory and damage responses, without the ability to restore functional myotubes. Additionally, ASO therapies often show limited delivery to heart tissue, and complications from cardiomyopathy are typically fatal in DMD patients (Nigro et al., 1990). Delivery of micro-dystrophin using adeno-associated viral (AAV) vector to express a functional, though shorter dystrophin protein, called micro-dystrophin, with preserved N- and C-termini in muscles is also an FDA-approved treatment for DMD, called ELEVIDYS. It utilizes a modified AAV, AAVrh.74 vector to deliver micro-dystrophin. This approach can offer long-term therapeutic efficacy by a single intravenous infusion. However, serious adverse effects have been confirmed, including acute liver failure, and two deaths in non-ambulatory DMD patients who received this treatment, in the March and June 2025 reports^1^. Finally, according to the request from FDA, Sarepta Therapeutics announced the voluntarily and temporarily pause shipments of ELEVIDYS for DMD in the US in July 2025^2^. Recently, an oral histone deacetylase inhibitor, givinostat, was approved by the FDA for the treatment of DMD patients aged 6 years and older. As with glucocorticoid treatment, this drug can potentially delay the progression of DMD but does not cure it. This has highlighted the need for additional therapies, which aim to promote the replacement of damaged tissue with healthy myoblasts that can successfully integrate into dystrophic tissue. Muscle stem cells (MuSCs), also known as satellite cells, have the potential to differentiate into mature myotubes if exposed to an optimal cocktail of cytokines, and offer a promising approach to replace degenerating tissue (Morgan et al., 1990; Partridge et al., 1989). However, simply injecting or grafting healthy stem cells into the diseased area is not enough to ensure complete regeneration due to the altered DMD patient muscle niche.

Significant challenges lie in the endurance of grafted cells, including survival of an elevated immune response, differentiation into myotubes in a fibrotic environment and protecting remaining dystrophic muscle between further treatments (Grounds et al., 2020; Kemaladewi et al., 2014; Monici et al., 2003; Pelosi et al., 2015; Tulangekar and Sztal, 2021; Villalta et al., 2011). To overcome these barriers, numerous strategies have been attempted. This includes triggering regeneration via stimulation of endogenous MuSCs, pharmacological or epigenetic pre-conditioning stem cells for enhanced engraftment and survival in a dystrophic muscle niche, post-operative stimulation to promote survival, and modulation of the immune response to reduce stress in grafted cells.

In this paper, we will summarize the endogenous mechanism for skeletal muscle repair and the inherent challenges facing researchers designing stem/progenitor cell-based therapies. We will highlight recent studies that seek to improve treatment of the skeletal and cardiac phenotype of DMD through cell replacement-based therapies. We will review the cell types thought to be most efficacious for grafting including myoblasts, MuSCs, CD133^+^ cells, mesenchymal stem cells, mesoangioblasts and dystrophin-expressing chimeric cells. We will particularly focus on pre- and post-treatment of cells to achieve better survival and summarize which treatments led to the most improved disease phenotype and entered clinical trials. Furthermore, we will highlight the various sources of healthy muscle stem cells, such as urine-derived stem cells and placenta-derived stem cells, evaluating the benefits and drawbacks of each.

## 2 Endogenous mechanisms of skeletal muscle regeneration

**FIGURE 3:**
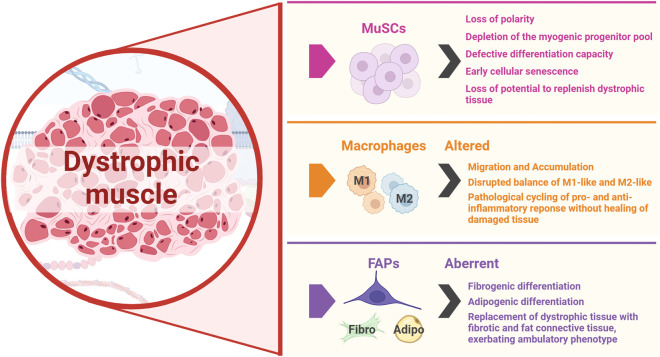
MuSCs, macrophages and FAPs are the key players in muscle regeneration whose activity is disrupted in DMD, both hindering repair and exacerbating disease progression. The figure was created with BioRender.com.

**FIGURE 4:**
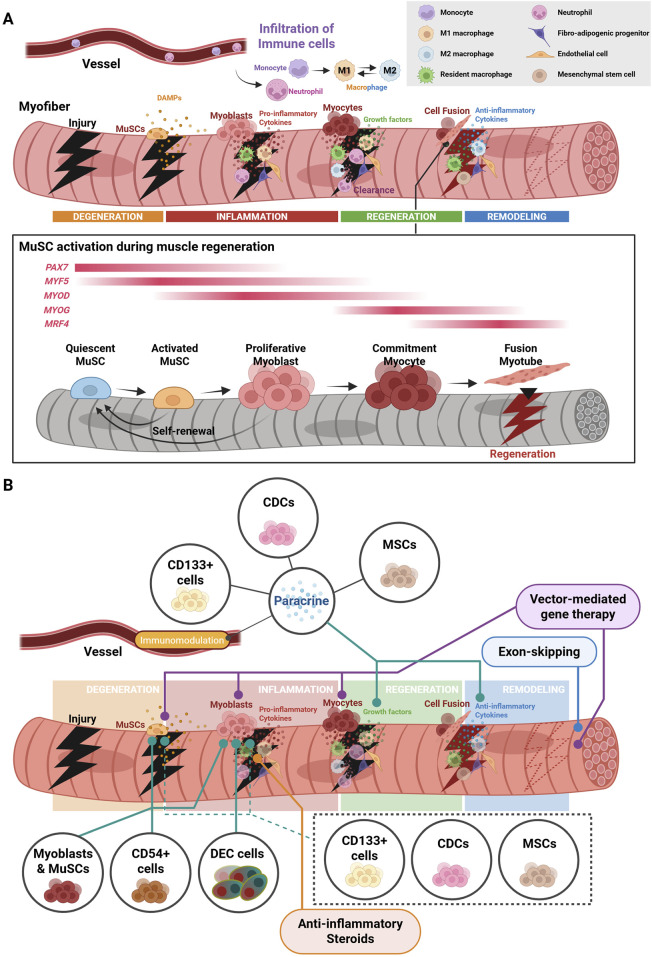
Acute injury results in a coordinated response towards regeneration pathways. **(A)** Muscle regeneration is triggered (1) upon a from the release of DAMPs from site of acute injury, (2) followed by a stepwise pro-inflammatory cytokine storm, (3) clearance and phagocytosis of necrotic tissue and debris, (4) and the invasion of anti-inflammatory macrophages that induce myogenesis and differentiation into matured myotubes from satellite cells. **(B)** Stem/progenitor cell and other cell-based therapies seek to target different stages of muscle repair. The figure was created with BioRender.com.

The cells of the muscle niche work in close interaction to maintain skeletal muscle tissue, but more crucially, have a strict, coordinated response in the context of stress or injury. This response is dysregulated due to the loss of dystrophin in DMD patients (Figure 1), leading to a progressively modified dystrophic muscle niche by several cells, such as muscle stem cells, macrophages, and fibro-adipogenic progenitors (Figure 3). Therapeutic intervention to restore muscle in these patients is highly challenging due to disrupted injury response and continuous cyclical aberrant immune responses (Tidball, 2017). Understanding of the stepwise mechanisms that are initiated during normal muscle regeneration is important to conceiving efficacious therapies, including the stimulation of newly generated tissue or grafted cells to successfully integrate into dystrophic muscle fibers and remain healthy. We will review the endogenous repair mechanism of skeletal muscle, along with triggers for MuSC activation and potential pathways that may be exploited for DMD muscle stem cell regeneration therapies (Figure 4A).

The degeneration phase is the initial response to muscle injury, characterized by the breakdown of damaged myofibers and the activation of muscle stem cells. Following acute injury, the affected tissues undergo necrosis, leading to the release of their cellular contents into the extracellular space along with damage-associated molecular patterns (DAMPs), such as myoglobin, ATP, mitochondrial nucleic acids, and high-mobility group box 1 (HMGB1) (Oishi and Manabe, 2018; Sciorati et al., 2016; Venereau et al., 2012; Zhang Q. et al., 2010). Certain markers of muscle damage, including creatine kinase (CK), are also released into the serum (de Freitas Nakata et al., 2021).

In the inflammation phase, DAMPs from necrotic fibers trigger the activation of resident macrophages into M1-like polarization, and the recruitment of circulating neutrophils and monocytes from the bloodstream (Tidball, 2017). Together, these cells coordinate an initial cytokine, pro-inflammatory response, where monocytes differentiate into M1-like macrophages and invade the damaged tissue (Arnold et al., 2007; Cheng et al., 2008; Lu, Huang, Ransohoff, et al., 2011; Lu, Huang, Saederup, et al., 2011; Payushina et al., 2019; Saclier et al., 2013; Tonkin et al., 2015). Several immune cells, including neutrophils and macrophages, play a role in the clearance of necrotic fiber debris via phagocytosis at the site of injury (Sciorati et al., 2016; Torres-Ruiz et al., 2023), while monocytes further differentiate into M1-like macrophages to sustain a pro-inflammatory immune response for a short time (Tidball, 2017). These infiltrated immune cells also secrete several growth factors, such as insulin growth factor-1 (IGF-1), as well as pro-inflammatory cytokines, creating a microenvironment conducive to regeneration (Bernard et al., 2022).

In the regeneration phase, activated MuSCs leave the quiescent state, enter cell cycle to proliferate and increase the pool of available progenitors (Wang and Zhou, 2022). The infiltrated M1-like macrophages in damaged muscle tissues shift to M2-like polarization through local cues (Varga et al., 2016). These cells coordinate the myogenic differentiation of the proliferating MuSCs to replenish lost myotubes through integration into host muscle. This is done through the expression of myogenic regulatory factors (MRFs), such as myoblast determination protein (MyoD), myogenic factor 5 (Myf5), myogenin, and MRF4 (Hernández-Hernández et al., 2017; Zammit, 2017). This differentiation is also partially regulated by resident mesenchymal progenitors called fibro-adipogenic progenitors (FAPs), which ordinarily play a role in remodeling of the extracellular matrix to provide for the MuSC niche (Chen W. et al., 2022; Uezumi et al., 2010), as well as providing support during muscle development. They also regulate inflammatory responses via interacting with immune cells, particularly macrophages (Brorson et al., 2024; Tidball, 2017).

The final phase involves the remodeling of regenerated muscle tissue, such as maturation, revascularization, and reinnervation of newly formed myofibers, restoring muscle function (Forcina et al., 2020). Resident and bone marrow (BM)-derived mesenchymal stem cells (MSCs) secrete growth factors and cytokines, supporting muscle regeneration (Andrade et al., 2015; Baglio et al., 2012; Fujita et al., 2015). Endothelial cells and pericytes (Koike et al., 2022) contribute to the revascularization, and Schwann cells (Guzman et al., 2023) (preprint) and guide cells (Ronzoni et al., 2021) contribute to the reinnervation. An extensive review of the existing literature on the diverse populations of immune cells triggered during injury, as well as DMD conditions, has already been covered in a previous report (Hernandez-Torres et al., 2024). We will instead focus on how these factors need to be considered when designing efficacious stem/progenitor cell-based therapies to treat DMD.

### 2.1 Muscle stem cells

The importance of insulin growth factor 1 (IGF-1) in muscle regeneration was identified in 1987, where MuSCs were found to be expressing this protein, particularly during regeneration (Hansson et al., 1987). Indeed, its expression in other regenerating tissues has also been identified (Dumont and Frenette, 2010; Ishii et al., 1994), suggesting a significant role through its regulation of myokine/cytokine Meteorin-like (Metrnl) – Stat3 activation (Baht et al., 2020). Regulation of the Notch pathway is key in the maintenance of the quiescent cell state. Healthy myofibers themselves, in addition to their function in movement and metabolism, interact with MuSCs to maintain quiescence under normal conditions. This occurs through direct N- and C-cadherin adhesive junction interactions, via the canonical Wnt-beta-catenin pathway (Madaro et al., 2019). Secreted factors are also crucial for maintaining this quiescence in the absence of injury or disease via YAP pathway repression and activation of the Rho-GTPase pathway.

Following activation by the immune system in injured muscles, Pax7^+^ MuSCs proliferate to expand the pool of progenitors, and asymmetric division occurs in parallel to generate myoblasts. However, lack of dystrophin and a robust DGC eradicates the necessary polarity and protein-protein interactions to drive this asymmetric division and inadvertently leads to deposition of fibrotic tissue (Duan et al., 2021; Madaro et al., 2019). As disease progresses, the affected niche becomes increasingly modified and is depleted of the crucial extracellular cues that maintain the MuSC population and diminish the interaction of macrophages and MuSCs.

### 2.2 Macrophages

The essential presence of a healthy immune response in muscle regeneration has been demonstrated through the ablation of macrophages or key pro-inflammatory factors using inducible mouse models (Giordano et al., 2015; Madaro et al., 2019; Nitahara-Kasahara et al., 2014). It was shown that resident MuSCs were redirected toward adipocytes instead of myoblasts and exhibited a reduced proliferative propensity, demonstrating the need for macrophages to promote and regulate stem cell maintenance and myogenesis (Madaro et al., 2019). Recovery of muscle from *mdx* ^*ITGAM-DTR*^ mice (*mdx* mice with transient macrophage depletion) following injury, and analysis by RNA sequencing showed that genes necessary for adipogenic differentiation were upregulated, and those for myogenic lineage were downregulated. This suggested that either the direct contact or secreted factors from macrophages are necessary to induce prolonged proliferation and promotion of the myogenic lineage. A similar mouse model that ablated MuSCs via conditional Cre-activated expression of diphtheria toxin receptor (DTR) in Pax7+ cells showed that muscle regeneration was significantly compromised following acute injury (Sambasivan et al., 2011). Therefore, strict cooperation between macrophages and MuSCs is necessary to establish the regeneration of myotubes.

Pro-inflammatory M1-like macrophages induce MuSC proliferation at the basal lamina, while macrophages invading the site of injury switch to an anti-inflammatory M2-like state and induce differentiation of myoblasts into myotubes. This switch is thought to be partially coordinated by an enzyme called sphingomyelinase (ASMase) that is upregulated in the middle stages of regeneration (Roux-Biejat et al., 2021). ASMase-KO mice exhibit a greater proliferation of MuSCs (CD34+/ɑ7-integrin+), accelerated regeneration, including increased muscle fiber diameter, and increased *Dmd* mRNA expression compared with wildtype controls. Additionally, the ratio of M1/M2 macrophages, as measured via flow cytometry of CD80 and CD206, was significantly reduced in ASMase-KO mice. Ablation of other components of this pathway, such as nuclear factor erythroid 2-related factor 2 (Nrf2), a transcription factor for anti-oxidative responses, have also shown complex responses in the regeneration process, including inhibition of the pro-inflammatory response, suggesting its elevation in this study may be compensatory (Bronisz-Budzyńska et al., 2020; Petrillo et al., 2017). This loss of balance between M1-and M2-like macrophages regulating muscle differentiation is a tightly regulated crosstalk, which could be challenging to replicate for therapeutic purposes. However, elevated regenerative response driven by ASMase-knockdown could be beneficial to restoring lost tissue in the short term, though further safety experiments are necessary to examine this (Roux-Biejat et al., 2021).

### 2.3 Fibro-adipogenic progenitors

FAPs are resident mesenchymal stem cells in the muscle niche with multilineage potential. They contribute to the support of other resident cells, as well as playing a role in crosstalk between the immune response and muscle cells during development and repair (Chen W. et al., 2022; Uezumi et al., 2010). Alterations to the immune response and niche in the context of DMD has implicated FAPs as a contributor to disease progression and present a significant challenge to overcome in therapy. This is due to sustained and abnormal activation, allowing infiltration of fibroblasts and adipocytes, leading to fibrosis. Ordinarily, FAPs have a transient role of support to the regenerative processes and enter apoptosis endogenously, clearing them and restoring the niche. This support includes triggering MuSC proliferation and myogenic cell fusion.

## 3 Mechanisms of muscle degeneration and defective regeneration in DMD

Dystrophin protein serves as a structural link between the muscle cell membrane and extracellular matrix via DGC, stabilizing the former during muscle contraction. Its absence results in increased membrane fragility and susceptibility to damage from mechanical loading. This membrane instability allows abnormal calcium influx, triggering proteolytic degradation and necrosis of myofibers (Dowling et al., 2022) (Figure 1B). Abnormal calcium influx in dystrophic muscle is driven by the dysregulation of multiple calcium ion channels, contributing to calcium overload and muscle degeneration. The store-operated calcium entry (SOCE) mediated by the complex of stromal interaction molecule 1 (STIM1), a calcium sensor in the sarcoplasmic reticulum (SR), and calcium release-activated calcium channel protein 1 (ORAI1), a plasma membrane channel, is hyperactivated in dystrophic muscle. This leads to disruption of calcium homeostasis and muscle damage (Goonasekera et al., 2014). The transient receptor potential (TRP) channels, particularly stretch-activated calcium channels TRPC1, TRPC3, TRPC6, and TRPV2, are overexpressed in dystrophic muscles, contributing to calcium overload (Gervásio et al., 2008; Harisseh et al., 2013; Vandebrouck et al., 2002). On the other hand, the ryanodine receptor 1 (RyR1), a calcium release channel in the SR, becomes dysfunctional by hypernitrosylation, resulting in the SR calcium leak (Bellinger et al., 2009). Together, these channel dysfunctions elevate cytosolic calcium, which activate proteases and muscle degeneration. Further aggravation of TRP and RyR channel activity is induced by excessive oxidative stress from an increase in reactive oxygen species (ROS). These abnormalities feed a vicious cycle in which mitochondrial dysfunction is amplified (Bonato et al., 2024).

As myofiber degeneration progresses, chronic inflammation and fibrotic/adipose tissues are observed in dystrophic muscle, which leads to impairments of muscle elasticity and function (Dowling et al., 2023). These DMD phenotypes are partly due to the dysfunction of several cell types, including MuSCs, immune cells, and FAPs, as well as abnormalities of calcium influx and mitochondria. DMD-MuSCs exhibit several features that lead to impaired muscle regeneration. Dystrophin deficiency downregulates microtubule affinity regulating kinase 2 (Mark2) expression, an enzyme with a key role in stabilizing mitotic spindle microtubule. This leads to a reduction in asymmetric cell divisions in MuSCs, where key components of the PAR complex cannot be phosphorylated and translocated to opposing ends of dividing cells (Dumont et al., 2015). Defective differentiation capacity in DMD-MuSCs occurs via alteration of cellular pathways, including reduced signal transducer and activator of transcription 3 (STAT3) (Liu Y. et al., 2025) and phosphatidylinositol-3 kinase (PI3K)/Akt signaling (Yazid and Hung-Chih, 2021). A decrease in the expression of MRFs leads to attenuated myogenic commitment (Esper et al., 2025). DMD-MuSCs exhibit early cellular senescence indicated by the enrichment of markers such as p16 and p19, with reduced activation and expansion potential (Cardone et al., 2023; Sugihara et al., 2020), and telomere shortening (Lund et al., 2007). Impaired muscle regeneration contributes to a progressively altered microenvironment in DMD muscles. Dystrophic muscles persist in aberrant production of pro-inflammatory and pro-fibrotic cytokines from dysfunctional MuSCs and accumulated mesenchymal progenitors (Cappellari et al., 2020). There is an elevated ratio of pro-inflammatory M1-like macrophages to anti-inflammatory M2-like macrophages (Hernandez-Torres et al., 2024), and disrupted interaction of MuSCs with macrophages and mesenchymal progenitors (Li Y. et al., 2025). These alterations contribute to chronic inflammation, fibrosis, and adipose tissue infiltration, which impairs muscle regeneration and induces muscle degeneration. Furthermore, an increasing number of studies are finding embryonic muscle developmental impairments associated with loss of dystrophin (Franzmeier et al., 2025; Kindler et al., 2025; Mozin et al., 2024). For example, somitogenesis is likely disrupted due to loss of dystrophin and the dysregulation of cell junction proteins, suggesting inherent deficits prior to birth (Mozin et al., 2024). This may suggest that MuSC replacement or replenishment therapies may be more beneficial at the earliest possible stage following diagnosis.

## 4 Current status of stem/progenitor cell-based therapies for DMD

### 4.1 Myoblasts and muscle stem cells

To date, stem/progenitor cell-based therapies for DMD have been investigated using numerous strategies, each offering potential benefits and challenges for restoring muscle function and slowing disease progression. In particular, the transplantation of MuSCs or myoblasts is a promising cell therapy for DMD to restore dystrophin expression and improve muscle function. In 1989, Partridge et al. reported that the intramuscular injection of normal myoblasts into *mdx* mice restores the expression of dystrophin and improves muscle function, as the transplanted myoblasts integrate with host myofibers (Partridge et al., 1989). The allogeneic transplantation of myoblasts into *mdx* mice consistently shows therapeutic efficacy against DMD muscle, including dystrophin restoration and functional improvements (Bourgeois Yoshioka et al., 2024; Kinoshita et al., 1994). However, the translation of these findings into human clinical trials have proved challenging.

In several double-blinded or not double-blinded clinical trials conducted in the 1990s, allogeneic intramuscular transplantation of 0.5–1.1 × 10^8^ normal myoblasts from DMD patients’ fathers or brothers into the biceps, tibialis anterior, or extensor digitorum longus muscle of 4 DMD patients aged 13–20 years (Huard et al., 1992), eight patients aged 6–10 years (Gussoni et al., 1992), eight patients aged 6–10 years (Karpati et al., 1993), 12 patients aged 5–9 years (Mendell et al., 1995), or 10 patients aged 5–10 years (Miller et al., 1997), showed a poor effect on dystrophin restoration and functional improvement compared to the placebo-injected contralateral muscle at up to 1 year after transplantation. Additionally, there are several limitations, including a loss of potency with *ex vivo* expansion and *in vivo* engraftments of transplanted cells (Biressi et al., 2020). A phase I/II clinical trial involving the transplantation of 3 × 10^7^ normal myoblasts per cm^3^ into the extensor carpi radialis of one forearm, with saline injected into the contralateral muscle as a control, in 10 DMD patients, was completed in January 2024 (NCT02196467). However, follow-up evaluation results for up to 6 months in this trial have not yet been reported. This likely suggests a significantly reduced propensity either for the transplanted cells to survive in the human disease niche, or for myogenic fusion of the grafted cells with surviving myotubes. Mice seem to demonstrate a much more robust ability to accept homologous cells and maintain their viability. Further studies into this specific process may help us to improve grafting patients with healthy myoblasts more efficaciously. The transplantation of MuSCs is considered more effective than that of myoblasts due to their stemness. The intramuscular allogeneic or xenogeneic transplantation of mouse or human MuSCs into *mdx* mice is efficiently engrafted into host myofibers, improving muscle function associated with an increased number of Pax7-positive mononuclear cells and dystrophin-positive myofibers (Arpke et al., 2013; Garcia et al., 2018; Sacco et al., 2008). Matre et al. reported that intramuscular transplantation of dystrophin-restored MuSCs derived from *mdx* muscles using CRISPR/Cas9 system results in an increase in dystrophin-positive myofibers and Pax7-positive cells at 4 weeks after transplantation (Matre et al., 2019). This approach allows for autologous cell transplantation to treat DMD. On the other hand, there are several limitations: the number of MuSCs isolated from muscle biopsies is very small, the engraftment and survival rate after transplantation are low, and MuSCs in the blood transplanted by the intravenous route are prone to aggregation (Sun et al., 2020).

Recently, cell transplantation utilizing human induced pluripotent stem cell (iPSC)-derived myogenic progenitors (MPs) has been widely studied for DMD treatment. Several studies successfully established a myogenic differentiation system from immature iPSCs through continuous expression of *MYOD1* (Goudenege et al., 2012; Tanaka et al., 2013) or *PAX7* (Darabi et al., 2012). He et al. conducted the transplantation of healthy donor iPSC-derived transgene-free MPs via the intramuscular route at 1 × 10^6^ cells, or the intravenous route at 2 × 10^6^ cells (He R. et al., 2020). Following intramuscular transplantation into *mdx* mice pretreated with immunosuppressant, iPSC-derived MPs are capable of integrating into the host myofibers, restoring dystrophin expression, improving muscle lesion, and repopulating the MuSC niche. Surprisingly, similar results are also observed in the intravenous transplantation of iPSC-derived MPs. Our group has reported the intramuscular transplantation of iPSC-derived MPs (1 × 10^5^ cells) generated by sphere-based culture into *NSG-mdx* ^4CV^ mice. Mice were treated with SB431542, transforming growth factor (TGF)-β receptor type I kinase inhibitor, to support myogenic differentiation from iPSC-derived MPs. Dystrophin-positive, human Lamin A/C-positive, and human spectrin-positive myofibers were consistently detected in the transplanted muscle (Sakai-Takemura et al., 2018). Miura et al. have established a method for iPSC-derived MuSC transplantation into diaphragm using hyaluronic acid-gelatin solution, resulting in the engraftment of iPSC-derived MuSCs in the diaphragm in *NOG-mdx* mice (Miura et al., 2022). These reports indicate iPSC-derived myogenic progenitors may be superior to myoblasts and MuSCs as transplantable cells.

Taken together, there is no doubt that the transplantation of myoblasts and MuSCs has promising potential for DMD treatment; however, there are still issues to overcome, such as the difficulty in obtaining the number of cells needed for transplantation while maintaining cell characteristics, low engraftment and viability rates, and tumorigenicity risk of iPSCs.

### 4.2 Dystrophin-expressing chimeric cells

Chimeric myoblast transplantation using dystrophin-expressing chimeric (DEC) cells is a unique therapeutic approach for treating DMD, as reported by Siemionow’s group. DEC cells are generated by polyethylene glycol (PEG)-mediated fusion of myoblasts from healthy and DMD-affected mice or human donors (Siemionow et al., 2018a; Siemionow et al., 2018b). The transplantation of DEC cells into *mdx* mice via systemic-intraosseous infusion increased dystrophin-positive myofibers, reduced inflammation and fibrosis, normalized myofiber diameters, and functional improvements in the cardiac, respiratory (diaphragm), and skeletal muscles at 90 and 180 days after transplantation (Siemionow et al., 2024; Siemionow et al., 2021; Siemionow et al., 2022). They reported that DECs possess mitochondria derived from healthy and DMD-affected donors and chimeric mitochondria created from healthy functional mitochondria and DMD-affected damaged mitochondria, indicating that the therapeutic effects of DECs are likely partially mediated by the delivery of functional mitochondria to DMD-affected muscles (Siemionow et al., 2024). In the first in-human trials, allogeneic myoblasts from healthy donors were fused with autologous myoblasts from DMD patients, called DT-DEC01, and delivered by a single systemic-intraosseous administration. Long-term follow-up showed no adverse effects for up to 22 months and improvement of skeletal muscle activity compared to baseline up to 12 months in ambulatory (n = 2) and non-ambulatory (n = 1) DMD patients (Niezgoda et al., 2023). On the other hand, while DEC cells reduce immune rejection due to their chimeric nature, allogeneic immune responses remain a safety concern. Since the efficiency of cell-to-cell fusion and dystrophin expression can vary, further optimization of the PEG-mediated fusion protocol is needed to improve therapeutic outcomes, and the number of patients in clinical trials is still insufficient. In addition, as with the transplantation of myoblasts or MuSCs, systemic delivery of DEC cells is challenging due to inefficient delivery to skeletal muscles. Dystrophin protein expression was also not analyzed in this trial. Despite these challenges, DEC therapy offers a novel and versatile solution for muscle regeneration in DMD patients.

### 4.3 Mesenchymal stem/stromal cells (MSCs)

#### 4.3.1 Paracrine and immunomodulatory properties of MSCs

MSCs are multipotent stem cells that can self-renew and differentiate into various cell types, such as osteoblasts, chondrocytes, adipocytes and myoblasts. Thus, MSC therapy has emerged as a promising therapeutic approach for several diseases, including DMD. The transplantation of MSCs derived from different tissues, such as bone marrow (BM), adipose tissue (AT), umbilical cord (UC), amniotic membrane (AM), and dental pulp (DP), in DMD model animals has been consistently associated with improved muscle function, regardless of the MSC origin. Mechanistically, MSCs are primarily involved in modulating muscle regeneration through paracrine mechanisms, rather than direct differentiation into myofibers and fusing with damaged myofibers. This includes inducing the proliferation and differentiation of MuSCs, reducing fibrosis, and stimulating angiogenesis by the secretion of soluble factors, such as cytokines, chemokines, and extracellular vesicles (EVs). Sandonà et al. have reported that intramuscular injection of EVs harvested from hAM-MSCs increases the number of activated Pax7^+^/Ki67^+^ MuSCs, stimulates myogenic differentiation, and reduces fibrosis in *mdx* muscles (Sandonà et al., 2023). Maeda et al. have reported that BM-MSC transplantation (2 × 10^6^ cells) in dystrophin/utrophin double-knockout mice by intraperitoneal injection stimulates the survival and function of MuSCs through secreting CXCL12, resulting in improvement of dystrophic pathology and extension of life span (Maeda et al., 2017). The intravenous transplantation of UC-MSCs (5 × 10^3^ to 5 × 10^5^ cells) has been reported to exhibit anti-fibrotic effects by secreting matrix metalloproteinase-1 (MMP-1) and microRNA 499 which targets TGFβR1 and TGFβR3, which promote muscle regeneration (Choi et al., 2020; Park et al., 2021). These reports strongly support the paracrine effects of MSCs in dystrophic muscles.

Another key feature of MSCs is their low immunogenicity, which allows them to evade immune responses. This is partly due to their low expression of major histocompatibility complex (MHC) class I and II molecules (Oh et al., 2022). MSCs are known to exhibit immunomodulatory effects. Intramuscular injection of AD-MSCs (1 × 10^6^ cells) stimulates M2-like polarization of macrophages, leading to a decrease in the pro-inflammatory cytokines such as TNF-α and IL-6 and an increase in anti-inflammatory cytokines such as IL-10 and TGF-β (da Pinheiro et al., 2012). The transplantation of hAM-MSCs (8 × 10^5^ cells) by intravenous tail vein route also induces M2-like macrophage polarization through the production of prostaglandin E2, improving dystrophic phenotypes in *mdx* mice (Nitahara-Kasahara et al., 2023). These immunomodulatory effects create a more favorable microenvironment for muscle regeneration.

#### 4.3.2 MSC-based combination therapies

Recent research has focused on combinatorial therapies, particularly MSC transplantation, to enhance therapeutic efficacy. Co-transplantation of MSCs with other stem/progenitor cells has shown promise in preclinical and clinical studies. Klimczak et al. reported that a co-transplantation of BM-MSCs and MuSCs directly into the dystrophic muscles of 3 DMD patients aged 11–22 years enhances electromyography parameters, reduces creatine kinase levels, normalizes pro-inflammatory cytokines, and restores dystrophin expression at up to 6 months after co-transplantation (Klimczak et al., 2020). Notably, in one patient who received co-transplantation with BM-MSCs and MuSCs in a 1:2 ratio (total 820 × 10^6^ cells), *DMD* mRNA expression was found to be 20% of normal and approximately 15% of myofibers were positive. On the other hand, one patient who received co-transplantation with BM-MSCs and MuSCs in a 1:10 ratio (total 700 × 10^6^ cells) showed a high level of systemic pro-inflammatory cytokines and no dystrophin-positive fibers were detected. Further examination is needed to optimize the ratio for co-transplantation, potentially even on a patient-to-patient basis.

The paracrine and immunomodulatory properties of MSCs support the engraftment and function of MuSCs to facilitate muscle regeneration. Vector-mediated gene therapy, particularly for mini- or micro-dystrophin expression, has been combined with MSC transplantation to overcome the limitations of each approach. Hayashita-Kinoh et al. reported the joint approach using MSCs and AAV-mediated gene therapy (Hayashita-Kinoh et al., 2021). In their strategy, the intravenous injection of hMSCs (2 × 10^6^ cells/kg body weight) mixed with rAAV9-carrying microdystrophin (5 × 10^5^ vg/cell) induced immune tolerance against rAAV9-microdystrophin in a dystrophic dog model, CXMD_J_. One week after the first injection, a second intravenous injection of MSCs was given, and the next day, rAAV9-microdystrophin was injected intravenously (2 × 10^12^ vg/kg body weight). As a result, this approach significantly improved the efficiency of gene transfer into dystrophic dogs, ameliorating dystrophic phenotypes.

Another study demonstrated that repeated systemic administration (10 times every 2 weeks) of DP-MSCs engineered to continuously express IL-10 (4 × 10^6^ cells/mL/kg body weight) in CXMD_J_, reduced muscle inflammation, promoted muscle regeneration, and improved locomotor activity by their potent immunomodulatory properties without adverse events up to 4 months after the last administration (Nitahara-Kasahara et al., 2021).

These combinatorial approaches aim to address the limitations of single-modality therapies by leveraging the complementary mechanisms of different therapeutic agents. While significant progress has been made in preclinical studies, further research is needed to optimize these therapies and translate them into effective treatments for DMD patients.

#### 4.3.3 Ongoing clinical studies of MSCs

Among several tissue-derived MSCs, clinical trials for UC-MSC therapies for DMD have been completed or are ongoing. Results from two phase 1 studies have been reported. Dai et al. performed transplantation of UC-MSCs at 2.5 × 10^6^ cells/kg following four intraarterial injections and four intramuscular injections in eight DMD patients. They showed an average increase of 17.6% (up to 60%) in *DMD* mRNA by real-time PCR and 20.1% (up to 38%) of dystrophin-positive myofibers by immunohistochemistry (NCT02484560) (Dai et al., 2018). The authors hypothesized that dystrophin restoration may result from cell-to-cell fusion of transplanted UC-MSCs with host DMD muscle cells, but this has not yet been proven in practice. The quantitative analysis of muscle strength by hand-held dynamometer measurements gave no significant difference, while electromyography showed an improvement between pre- and post-transplantation. Another phase 1 clinical trial using UC-MSCs (EN001) determined that the treatment of EN001 at low doses (5.0 × 10^5^ cells/kg) and high doses (2.5 × 10^6^ cells/kg) was safe and well-tolerated in six DMD patients aged 10–16 years, with no severe adverse events during the 12-week follow-up period (Lee et al., 2025). Although the muscle functional tests, including myometry measurement, North Star Ambulatory Assessment (NSAA), and 6-min walk test (6MWT) observed no significant changes from baseline in short-term functional evaluations, a future clinical trial is planned to evaluate the safety and efficacy of EN001 during long-term follow-up in a multi-center, double-blind, placebo-controlled phase 1/2 trial (NCT06328725).

### 4.4 Deramiocel: cardiosphere-derived cells (CDCs)

Cardiosphere-derived cells (CDCs), cardiac progenitor cells derived from cardiac tissue, are expected to improve both cardiac and skeletal muscle function in DMD. Similar to MSC transplantation, the therapeutic effects of CDCs are primarily mediated through paracrine mechanisms. Aminzadeh et al. have reported that intramyocardial injection of CDCs or CDC-derived exosomes improved DMD pathology of the heart, soleus muscle, and diaphragm in *mdx* mice, as well as improved muscle growth (Aminzadeh et al., 2018). Surprisingly, they showed that the expression of full-length dystrophin protein in the heart, soleus, and diaphragm is transiently restored after injection of either CDC or CDC-derived exosomes. This mechanism is thought to be mediated by miRNA-148a contained in CDC-derived exosomes (Ibrahim et al., 2014). However, this restoration was only observed after 1–3 weeks following treatment in both CDC and CDC-exosome treatments and had dissipated by 3 months, showing the need for other strategies for prolonged dystrophin expression. The same group later reported that a single intravenous dose of CDCs in *mdx* mice positively impacts cardiac and skeletal muscle function by reversing heart damage and enhancing skeletal muscle regeneration, partially through the paracrine effect of exosome secretion (Rogers et al., 2019). Notably, the therapeutic effects of CDCs were not mediated by restored expression of full-length dystrophin, since its expression was less than 0.5% of wildtype levels (Rogers et al., 2019). While optimistic, it should be noted that there was an elevation of stress factors following the injection of CDCs into *mdx* heart. Specifically, factors that respond to mitigate inflammatory and oxidative stress, such as phospho-AKT and nuclear NRF2, were elevated highlighting a potential post-operative side-effect that may not be suitable for later-stage patients (Aminzadeh et al., 2018). The overall number of CD68^+^ and CD3^+^ immune cells was reduced 3-week following treatment, suggesting this effect is responsible for clearing the disease aberrant response, though whether this persists long-term is less clear. The effect of using CDC-derived exosomes to reproduce some of the benefits of CDCs was investigated, showing reduced fibrosis (lower collagen type I A1 and type III A1). Finally, significant extended lifespan of the CDC-treated mice was demonstrated, where over 50% survived up to 23 months, by which time all in the vehicle-treated group had died. The benefits of exosomes-derived from CDCs are evident, but there are several factors that warrant more in-depth focus. This includes the potential side effects from these exosomes reaching brain, liver, spleen, gut and kidneys, particularly due to repeated injections over a long-term basis (Aminzadeh et al., 2018). Additionally, there remains a significant challenge in the large-scale production of exosomes for therapy. This therapy will likely require large cultures of CDCs to harvest exosomes, combined with the need for stringent quality control, as a potential therapeutic agent is likely to be both expensive and labor-intensive.

These combined results led to the design of the HOPE-Duchenne (Halt cardiomyopathy progression in Duchenne) trial (NCT02485938), a phase I/II study to demonstrate the feasibility, safety, and efficacy of intracoronary allogeneic CDCs in patients with cardiomyopathy secondary to DMD (Ascheim and Jefferies, 2016). Taylor et al. have shown that intracoronary infusion of Deramiocel (CAP-1002), a clinical formulation of human allogeneic CDCs, improves cardiac and upper limb function in 13 DMD patients with myocardial fibrosis compared to 11 control DMD patients for up to 12 months (Taylor et al., 2019). In the HOPE-2 phase II trial (NCT03406780), intravenous infusions of Deramiocel (once every 3 months for a total of four infusions) in ambulatory or non-ambulatory DMD patients improved limb and cardiac functions (McDonald et al., 2022). Further open-label extension of the HOPE-2 trial (HOPE-2-OLE), which provides Deramiocel to DMD patients enrolled in the HOPE-2 trial and will complete 12 months of follow-up, is ongoing (NCT04428476). In 2025, the FDA approved Deramiocel and granted it priority review for DMD cardiomyopathy. This trial has shown improvement of skeletal muscle symptoms in DMD patients, but due to a limit of 12-month follow-up, verification of sustained upper limb improvements or whether repeated treatment replicated more of the pre-clinical phenotypes, could not be validated. Further, restoration of dystrophin through biopsy examination of skeletal muscle tissue, was not examined this time. Future reviews should revisit these questions and consider monitoring patient cognitive behavior for any off-target paracrine signaling effects.

### 4.5 Mesoangioblasts

Mesoangioblasts are multipotent vessel-associated stem cells originally isolated from the dorsal aorta of quail and murine embryos (Minasi et al., 2002). Mesoangioblasts have dual roles in crossing blood vessel walls and differentiating into various mesodermal cell types. As with MSCs, mesoangioblasts exhibit immunomodulatory effects, suppressing T-cell proliferation through inoleamine 2,3-dioxygenase and PGE2 pathways (English et al., 2013). Serena et al. reported that human primary mesoangioblasts restored dystrophin and distribution by cell-cell fusion via an *in vitro* model of human DMD skeletal muscle tissue-on-a-chip more efficiently than human myoblasts (Serena et al., 2016). *In vivo* studies have shown that the transplantation of mesoangioblasts improves dystrophic pathology in *mdx utrn* ^*−/−*^ mice and a Golden retriever muscular dystrophy model (GRMD) (Berry et al., 2007; Sampaolesi et al., 2006). Using a combination of mesoangioblast transplantation with other approaches, Tedesco et al. reported that mesoangioblasts from *mdx* mice are genetically corrected with a human artificial chromosome (HAC) vector containing the whole human dystrophin gene (2.4 Mb). Intramuscular or intra-arterial injection of these corrected mesoangioblasts into immune-deficient *mdx* mice resulted in cellular engraftment in dystrophic muscles with restored dystrophin expression and improvement of muscle function (Tedesco et al., 2011). An intriguing finding from these studies was that cells derived from the patient, corrected and transplanted back, was far less efficient at restoring dystrophin than injected cells from healthy donors. This is likely to represent a hurdle in future studies where appropriate human leukocyte antigen (HLA)-matched donors will need to be identified for each patient. This could prove detrimental in terms of therapeutic access for a large number of patients by increasing waiting times, particularly where multiple treatments will be required over the course of their disease progression.

The first in-human clinical trial (phase I/II) of intra-arterial mesoangioblast transplantation in DMD patients demonstrated safety and feasibility (Eudract 2011–000176–33); however, the therapeutic efficacy was limited, as evident by transient functional stabilization and poor engraftment of cells (Cossu et al., 2015). Furthermore, the cohort of individuals was low, at five DMD patients receiving the transplanted cells. While the key aim of this trial was to evaluate safety, the patients exhibited a range of different symptoms during and after the trial, making interpretation of their response to the therapy challenging. Some side effects following infusion of the mesoangioblasts included *Livedo reticularis* in two patients and acute small thalamic stroke in one patient (Cossu et al., 2015). While the latter did not present with any clinical consequences, due to the small cohort and relatively higher degree of side effects in three out of five participants, it is hard to conclude on the effect of this therapy on a larger group. The initial animal studies were compelling to proceed with this method for human trials, though a number of technical barriers remain towards seeing a clear clinical improvement via mesoangioblast infusion therapy.

### 4.6 CD133^+^ cells

CD133^+^ cells, a subpopulation of stem cells, have shown significant potential for treating DMD. CD133^+^ cells are an accessible option for autologous cell therapy because they can be isolated from various sources, such as peripheral blood, bone marrow, umbilical cord and muscle tissue (Meregalli et al., 2012). Torrente et al. have reported that when blood-derived circulating CD133+ cells are cocultured with mouse myogenic cells or fibroblasts expressing Wnt7a, they can fuse with mouse myogenic cells to form multinucleated myotubes (Torrente et al., 2004). The transplantation of CD133^+^ cells into immunodeficient *scid-mdx* mice via three intramuscular injections (2 × 10^4^ cells each) or intra-arterial delivery (5 × 10^5^ cells) resulted in the formation of dystrophin-positive hybrid regenerated fibers, especially in intramuscular transplants. Additionally, functional recovery through single myofiber analysis was observed in dystrophic muscle transplanted with CD133^+^ cells. CD133^+^ cells also secrete microvesicles expressing several pro-angiogenic and anti-apoptotic factors, including IGF-1, VEGF and IL-8, promoting a regenerative microenvironment through paracrine effects (Ratajczak et al., 2013). In one phase I, double-blind clinical trial study, the autologous transplantation of muscle-derived CD133^+^ cells proved safe, but no effective integration into host muscle nor muscle strength improvement was seen at a 7-month follow-up (Torrente et al., 2007). The reason for 7 months as the determined endpoint for the trial is unclear, with no intermediate time point evaluations, though no adverse effects were reported by any participants. This therapeutic strategy represents a significant gap between pre-clinical observations in animals and the human trial. A significant amount of work is yet needed to evaluate its efficacy and optimize conditions for successful survival and integration into human patient tissue. Further investigation, paired with more recent findings related to the pre-treatment of cells for grafting may improve this modality.

### 4.7 MyoPAXon: iPSC-derived CD54^+^ myogenic progenitors

MyoPAXon, developed by Dr. Perlingeiro’s team and Myogenica, is myogenic progenitors generated from human umbilical cord or a cGMP-certified iPSC-derived myogenic platform^2^. The following paper reported by Dr. Perlingeiro’s team is likely the source of MyoPAXon product, though a clear origin is not cited. They demonstrated that the intramuscular transplantation of iPSC-derived PAX7^+^ myogenic progenitors expressing CD54, integrin α9β1, and syndecan 2, in DMD model *NSG-mdx* ^*4cv*^ mice shows long-term engraftment (up to 10 months) (Magli et al., 2017). Additionally, in preclinical studies using animals, the intramuscular, intravascular, and intra-arterial transplantation of MyoPAXon showed safety, engraftment in muscles and functional improvement (Azzag et al., 2025). The phase 1 trial is currently recruiting patients and is scheduled to begin in 2025 (NCT06692426).

### 4.8 Gene-edited cells

The majority of cell therapies thus far have described studies using wildtype cells that readily express dystrophin to enhance grafting of healthy stem cell populations in treated animals and improve local myotube integration. However, in these cases the need to identify allogeneic matches for patient grafts poses a significant challenge. Instead, taking the patient’s own cells, correcting or partially correcting dystrophin expression via a CRISPR-guided strategy, and restoring them via transplantation may be an alternative method to overcoming this challenge. Multiple methods to screen for the optimal ASOs to improve dystrophin protein expression through exon-skipping and delivery of mini- or micro-dystrophin mRNA have been attempted, though levels of restored protein remain insufficient for phenotypic improvement. Additionally, many of these therapies aim towards directly targeting degrading myotubes, or a significantly depleted MuSC population in the patient through systemic injections. Fewer studies have shown transplantation of patient corrected cells into patients or a disease model, likely due to relatively lower success rates. As such, this is an area that remains open for deeper investigation. The original study to trial this method was by Moisset and colleagues. This study corrected an *ex vivo* biopsy from a DMD patient via adenoviral transduction of human mini-dystrophin gene, followed by transplantation into the TA of an injured, immunosuppressed WT mouse (Moisset et al., 1998).

While they have not all been applied *in vivo*, an increasing number of publications using novel CRISPR-based systems are being developed that have a special interest in the DMD field as they can target a significant proportion of the patient population (Echigoya et al., 2018; Kita et al., 2023; Laurent et al., 2024; Li H. L. et al., 2015; Ousterout et al., 2015; Young et al., 2016). Kita et al. recently demonstrated the induction of a multi-exon skipping system, which could potentially be applied to over 60% of patients (Kita et al., 2023). This method utilized a dual CRISPR-Cas3 system to optimize editing of exon 45–55 (almost 350 kb) and restoring the reading frame of the DMD gene. This editing strategy would allow the expression of a significantly truncated, but partially functional dystrophin protein, that could extend lifespan significantly if successfully delivered. This study focused on optimizing the system but also demonstrated that dystrophin protein could be restored in various DMD patient-derived iPSC lines. Though no *in vivo* data was included here, another study performed exon 45–55 correction in an immortalized patient-myoblast line with an exon 48–50 deletion. The out-of-frame mutation was corrected in these cells and subsequently transplanted into immunodeficient NOD. SCID.gamma mice, though a relatively low number of human dystrophin-positive fibers could be detected (Ousterout et al., 2015). This shows that while there is potential for wide patient applicability via the same guide sequences and concerns for immune reaction are lessened, the integration of myoblast-derived grafts may be poor and iPSC-derived grafts are unclear. Additionally, there is estimated to be a long and expensive route between collection of patients cells to transplantation. This includes culture expansion and performing reprogramming, inducing multi-exon skipping and quality control preparation for surgical transplantation.

Another study has explored the potential of a peptide nucleic acid single-stranded oligodeoxynucleotide (PNA-ssODN) to perform single-base DNA site-directed gene editing to permanently restore dystrophin in harvested MuSCs from a *mdx* ^*5cv*^ model mouse (Bertoni et al., 2005; Kayali et al., 2010; Nik-Ahd and Bertoni, 2014). While the correction of the mutation via this method is high and transplanted cells into the mouse demonstrated a decent percentage of dystrophin positive fibers in the TA muscle up to 24 weeks after transplantation, this method can only be applied to DMD patients with single base mutations in the DMD gene. Though this is a relatively small proportion of patients, increasing interest in developing patient-tailored treatments may provide a route for using this method for a niche patient subset.

Finally, muscle- or blood-derived CD133+ cells have been investigated as a source of patient cells for engineered correction (Benchaouir et al., 2007). Here, lentiviral vectors were used to express ASOs to induce exon skipping, and by the intramuscular route into *scid/mdx* mice. Interestingly, human dystrophin was partially restored in TA 21 days post-transplantation, with more positive fibers after 45 days, though muscle-derived CD133+-corrected cells showed up to a 4-fold increase compared with blood-derived cells. This may highlight an importance for considering lineage when obtaining cells for grafting. Muscle-derived corrected cells were then examined for engraftment via intra-arterial injection, and while it was not as efficacious as the former, some dystrophin-positive fibers were also seen in quadriceps and soleus after 21 days (Benchaouir et al., 2007). Some improvement in motor function was observed including normalized tetanic force and in the treadmill test, though it was unclear how long these were sustained. Overall, proof-of-principle has been demonstrated for correction of patient-derived cells followed by grafting in myoblasts and CD133+ cells, though has yet to be translated into DMD patients.

## 5 Improving the efficacy of stem/progenitor cell-based therapies and challenges

The precise reason for poor translation from pre-clinical mouse studies to human trials is under-investigated, though it may be linked to the discrepancy in disease pathology between murine models and humans, or glucocorticoid treatments in patients. In the case of the latter, glucocorticoids, such as prednisolone, are used to treat patients to delay loss-of-ambulation by suppressing the cyclical pro- and anti-inflammatory immune response. However, these treatments may cause adverse side effects, which may impede cell-based therapies, though strong direct evidence for this is lacking. For example, prednisolone targets the NF-κB pathway, leading to the downregulation of IL-12, IL-6 and TNF-α (cytokines with complex roles in immune response) (Blotta M H et al., 1997; DeKruyff et al., 1998; Liu G. et al., 2024), inhibits macrophage activation and downregulates the expression of cellular adhesion molecules (Villalta et al., 2009; Wehling-Henricks et al., 2004), all of which could affect the muscle niche and alter the potential for myogenic regeneration. Alterations to the immune response may have deleterious effects on transplanted cells to survive and integrate into the disease niche. Furthermore, a study using C2C12 cells found that IL-12 enhanced myogenic differentiation in this *in vitro* model, suggesting that some steroid treatments may impede regeneration even with the introduction of healthy cells (Romanazzo et al., 2015). Conversely, while these treatments frequently cause cytokine inhibition, IL-12 elevation in diaphragm has also been detected following spironolactone and prednisolone treatment in *mdx* mice (Howard et al., 2022). Overall, we cannot conclude a definitive mechanistic explanation for the poor efficacy of cell-based therapies between humans and murine models, though we may consider more thoroughly investigating their efficacy when combined with patient therapeutic history.

### 5.1 Pre-conditioning of stem/progenitor cells to promote muscle integration

Pre-conditioning of stem/progenitor cells is a promising strategy to enhance their integration into muscles for treating DMD. This involves biochemical or environmental priming of stem/progenitor cells before transplantation to improve post-operative survival and functional integration into host muscles. Various preconditioning methods have been studied, including multimodal treatments.

Epigenetic profiles of healthy regenerating muscle and DMD epigenomes have been examined in depth to clearly understand the molecular modifications that may help correct the disrupted differentiation of MuSCs into myotubes. However, the design of a clinical therapy is relatively recent (Aartsma-Rus, 2024; Mozzetta et al., 2024; Rugowska et al., 2021). Myogenic progenitors reprogrammed from iPSCs using *givinostat*, a histone deacetylase inhibitor approved by the FDA to treat DMD, exhibit enhanced proliferative and migratory traits compared to untreated controls and normal adult myoblasts (Xuan et al., 2021). The iPSCs were initially treated with CHIR99021, a glycogen synthase kinase-3 (GSK-3) inhibitor, for myogenic lineage induction, followed by *givinostat*, to make ‘Givi-MPC’. The transplantation of Givi-MPC into the injured muscle of *mdx/SCID* mice showed minimal necrosis, reactive oxidative species, and inflammatory response, but pro-angiogenic activity was observed. Crucially, dystrophin protein was more significantly restored compared to injection of human myoblasts without *givinostat* treatment, with the formation of new BTX-positive neuromuscular junctions, suggesting functional integration (Xuan et al., 2021). Most importantly, 2 months following the initial transplantation, another injury was induced, and analysis of the muscle 1 month subsequently revealed that the Givi-MPCs had survived and repopulated the muscle niche. Finally, established Givi-MPC secreted exosomes that were enriched for pro-angiogenesis factors relative to untreated adult human myoblasts, demonstrating the importance of paracrine/endocrine signaling at the muscle niche for regeneration, in addition to the survival of healthy, dystrophin-expressing cells (Xuan et al., 2021). This has also been demonstrated through intramuscular injections of stem/progenitor cell-derived secretome factors, which showed the ability to enhance muscle cell growth and reduced lipid deposition in mice over 2 years old (Fennel et al., 2024). The components of stem/progenitor cell secretome involved in tissue remodeling and promoting cell growth are likely crucial for improving overall healing and regeneration in tissue. While it is unclear why direct transplantation of healthy MuSCs into human patients is markedly less successful compared with pre-clinical animal trials, a potential consideration may be given to the dampening of cell-autologous signaling to promote their integration into the dystrophin muscle niche. To improve stem/progenitor cell-based therapies, enhancement of the conditions for regeneration may be applied via cocktails of regenerative factors. Thus, further studies to better understand these factors is crucial.

Another study found that the decreased myogenicity of DMD patient biopsy-derived MuSCs was associated with the dysregulation of epigenetic regulatory factors, such as chromobox protein homolog 3 (CBX3), histone variants H2AZ2 and H3F3B, and structural maintenance of chromosomes 3 (SMC3). Restoration of these factors via lentiviral plasmid delivery improved myogenic differentiation potential *in vitro* (Massenet et al., 2024). While likely to be highly challenging to deliver multiple similar plasmids *in vivo* for therapy, the modulation of their downstream elements, such as myocyte enhancer factor 2B (MEF2B), may improve local myogenic differentiation in combination with stem/progenitor cell therapies.

Previous reports have demonstrated the efficacy of BM-MSCs in elevating myoblast survival and engraftment, and the potential for secreted exosomes as a replacement for direct treatment through injection of these cells for therapy (Archacka et al., 2021). Optimization of such pretreatments for muscle disease may improve integration of stem/progenitor cells without the need to prepare and inject multiple cell types at the site of damage (Kim et al., 2016; Lin et al., 2016). Guo and colleagues have reported that repeated intramuscular injections of exosomes from BM-MSCs cultured in hypoxic conditions promote MuSC activation and muscle regeneration through a miR-210-3p-mediated mechanism in aged rats (Guo et al., 2024). This preconditioning was a vital step in inducing the BMSCs into secreting miR-210–3p, whose knockdown in the exosomes diminished their conferred benefit. MiRNA-210–3p is upregulated in response to hypoxia, triggering the activation of pathways associated with vascularization and mitochondrial function (He N. et al., 2020), hence its potential to positively modulate the muscle niche in dystrophic muscle, where this is disrupted. Although this work was done in an ageing rat model rather than a DMD one, the potential to treat healthy engrafted cells for improved integration is evident.

Pretreatment of muscle stem cells under hypoxia with interferon-*γ*, prior to transplantation, has also been shown to be beneficial to jointly improve stem cell viability and prevent elevated CD4^+^/CD8^+^ T cell invasion. This was linked to the upregulation of Programmed Death Ligand 1 (PD-L1) in these cells, whose increased expression correlated with improved survival of injected MuSCs in the gastrocnemius muscle of adult *mdx* mice (Raiten et al., 2024). PD-L1 itself was upregulated via exposure to hypoxic conditions. Conversely, knocking down PD-L1 significantly reduced the survival of the grafted cells, while interferon-*γ* was crucial to minimizing T cell activation. Similarly, inducing oxidative stress has been used as a method for clonal selection, making them better candidates for transplantation (Gargioli et al., 2018). Together, these studies highlight a key pretreatment for the survival of grafted cells through pre-selection in stressed conditions.

### 5.2 Enhancement of cell viability post-engraftment

In addition to improving the viability of grafted healthy cells, significant progress has also been made in extending the viability of DMD muscle tissue. DMD muscle cells exhibit numerous metabolic phenotypes, including elevated calcium-induced apoptosis and mitochondrial deficits, which further impede the function of degenerating myotubes (Alderton and Steinhardt, 2000; Allen et al., 2015; Mareedu et al., 2021). Treatment of dystrophic muscle to address these phenotypes before and after treatment with cell-based modalities may improve the longevity of the tissue and potentially increase the chance of integration of grafted healthy cells. Specifically, 2-aminoethoxydiphenyl borate (APB) has been shown to mitigate the overload of Ca^2+^ in the dystrophic niche by inhibition of multiple calcium channels (Dubinin et al., 2024; Mondin et al., 2009). *In vivo*, daily administration of 3 mg/kg APB after 4 weeks showed a slight decrease in serum creatine kinase, and reduced fibrosis in the quadriceps of *mdx* mice, though not sufficient to restore grip strength. However, the investigators did not observe a decrease in aspartate aminotransferase or lactate dehydrogenase (Dubinin et al., 2024). Clinical use of this drug may be beneficial in slowing disease progression, though it still requires significant investigation, particularly regarding the appropriate dosage for beneficial treatment. Mice with similar daily treatment of 10 mg/kg APB did not show improved muscle function either, though they had a reduced number of mitochondria, of which the remaining ones had serious morphological alterations, including swelling and deformed and fragmented cristae, as identified by electron microscopy. While mild phenotypic improvements in 3 mg/kg-*treated mdx* mice could be attributed to APB-induced Ca^2+^ homeostasis, it is estimated that the wide range of calcium modulators targeted by this drug are likely to cause more harmful off-target effects. Instead, screening for other candidates that target a similar mechanism may be warranted, such as specific modulators of IP_3_ receptor (Valladares et al., 2018). Nevertheless, a more metabolically balanced niche is likely to allow newly induced healthy control stem cells to integrate into regenerating DMD muscle. Significantly, Dubinin et al. showed that treating wild-type mice with ABP did not impair their muscle function or increase serum biomarkers for damage, meaning that this kind of co-therapy is unlikely to hinder their survival.

Improvement of DMD metabolic symptoms has also been examined through the activation of an NAD^+^-dependent deacetylase, Silent information regulator 1 (SIRT1). This protein triggers mitochondrial biogenesis, fatty acid oxidation, and anti-inflammatory activity in muscle tissue in response to events that increase cellular energy demand (Fischer et al., 2009; Gerhart-Hines et al., 2007; Singh and Ubaid, 2020). As a stress-sensing protein, increasing its activity may help mitigate compromised metabolic activity in dystrophic muscle. Oral administration of SIRT2104, a selective SIRT1 activator, to DMD models of *Drosophila melanogaster* (Dys^E17^) and mouse (*mdx)* demonstrated improved muscle morphology and function (Giovarelli et al., 2025). In mice, 100 mg/kg of SIRT2104 was supplemented to the food daily for 12 weeks from 8-week-old *mdx* mice. Additionally, treated *mdx* tissue exhibited significantly decreased CD45^+^ immune cell invasion, myonecrotic fibers, and fibrosis. Interestingly, the SIRT2104-treated mice also exhibited an upregulation of embryonic myosin heavy chain (MyHC-Emb), suggesting activated regeneration. Improved ATP bioavailability was identified through proteomic analysis. Mitigation of dystrophic tissue both before and after engraftment with SIRT2104, or other SIRT1 modulators, might be key to enhancing the inclusion of healthy stranger cells both through improved metabolic management and inhibiting the immune response. The presence of readily regenerating embryonic cells may help sustain the niche for therapeutic MuSC integration, though further investigation of this through a combinatorial therapeutic strategy is needed. SIRT2104 was previously involved in a diabetes Phase I/II trial and passed safety evaluation, but since has been excluded from further investigation for this disease and has not been approved by the FDA for human-based therapy (Baksi et al., 2014). However, it exhibited promising activity in earlier studies from improving cognitive health through modulation of the SIRT1 pathway in the brain of a diabetes-induced cognitive dysfunction mouse model, and remains promising for DMD therapy (Yang et al., 2021).

Reduced physical activity in DMD patients contributing to immobilization-induced muscle atrophy, chiefly driven by FAPs, is another barrier that may impact the efficacy of stem/progenitor cell based-therapies. Assisted physiotherapy may help minimize this phenotype, in addition to activation of IL-33-ST2 signaling (Takahashi et al., 2024). Originally designed to help ageing individuals with an increasingly sedentary lifestyle, it is unclear if targeting this pathway would be relevant in younger DMD patients. Specific exercise regimens have been investigated to minimize the progression of muscle dystrophy in DMD patients (Yamauchi et al., 2025), though they are not yet routinely included in clinical trials in conjunction with transplanted stem/progenitor cells to encourage regeneration.

A novel method of improving grafting and survival of transplanted cells has recently been developed through the embedding of CRISPR/Cas9-corrected DMD patient iPSC-derived myogenic progenitor cells in a safety-tested hydrogel, and surgical transplantation of a 5 mm cell-laden construct into an immunodeficient mdx mouse (Kowala et al., 2025). While, no functional recovery information is available due to the lack of motor tests at the 6-month point of analysis, the human origin cells replenished resident MuSC populations and the myofibers exhibited innervation and vascularization, suggesting that late-stage regeneration phases were being fulfilled. Furthermore, tumorigenesis was not observed, passing a major safety step. This method is able to safely carry and maintain longevity of cells, where single cell grafts often fail, particularly in human trials. Another recent innovation was the generation of 3D mouse skeletal muscle organoids, from which *in vitro*-derived satellite cells emerged to play a role in regeneration following grafting onto both injured and DMD mouse (*mdx* ^*5cv*^) tissue (Price et al., 2024). Using normal myoblast grafts as a control, a multi-fold increase in the number of grafted cells (using a tdTomato reporter) was demonstrated in the injured wildtype model, with almost 60% of TdTom + fibers following re-injury. Though, the *mdx* ^*5cv*^ model only showed similar recovery to injected satellite cells initially, ∼20% of dystrophin+/laminin + fibers were quantified followed re-injury, showing a considerable survival rate in the long term. Thus, an organoid model may be suitable both as a delivery system and self-contained niche for the transplantation of MuSCs in disease. Both novel therapies yet lack improved functional phenotype in the animals, but they represent new directions for in depth investigation with many points of optimization that may render them suitable for human therapy. This includes from a surgical perspective and the avenue of testing many hydrogel or artificial scaffolds which may be purpose-built for DMD tissue endurance.

### 5.3 Challenges of stem/progenitor cell therapies

Stem/progenitor cell therapies have proven relatively effective in clinical trials, with modest side effects and rare severe adverse events, however persistent challenges remain in achieving approval for DMD treatment.

Immune rejection is a major hurdle for all stem/progenitor cell therapies, whether using autologous cells where severe reactions can be avoided but not completely, or allogeneic which is limited by diminished therapeutic efficacy. Systemic delivery is also challenging. Most cells injected into the bloodstream are sequestered to the lungs, liver, and spleen (Fischer et al., 2009), with very few cells can reach the muscle tissue. Several cell types, including DEC cells, CDC cells, MSCs and mesoangioblasts have shown clinical outcomes with systemic delivery in DMD patients. However, their effect has often been attributed to paracrine effects, and replenishment of dystrophin-positive myofibers has not been sufficiently achieved. Additionally, the aggravated environment of dystrophic muscle tissues, such as inflammation, fibrosis, and fat infiltration, can shock the transplanted cells from *in vitro* conditions, leading to poor cell engraftment and survival. Overcoming regulatory hurdles of stem/progenitor cell therapies requires concrete evidence for long-term safety and therapeutic efficacy, a robust systemic delivery system and manufacturing management (standardizing and scalability). In addition, obtaining informed consent from patients who understand the potential uses and implications of their cells and protecting the their personal information are critical to addressing ethical issues. It is also important to provide opportunities for communication and discussion with the public, physicians/researchers, and policy workers by preparing a forum for disseminating and sharing information to society.

The scalability of stem/progenitor cell therapies for DMD patients presents manufacturing and financial hurdles. Preparing billions or trillions of clinical-grade cells for transplantation from a single patient or donor that ensures high-quality, high-purity, safety, and efficacy requirements is challenging. In the case of autologous transplantation, preparing a patient-specific (personalized) cell batch makes standardization nearly impossible due to the time-consumed and high cost. On the other hand, with allogeneic transplantation, scaling cells up from a single donor can provide allogeneic transplantation for many patients but requires massive bioreactors and vast donor screening to avoid the risk of immune rejection. There is an urgent need to establish cell banks that comply with the current Good Manufacturing Practices (cGMP) grade. In addition, stem cells with genetic correction, such as iPSCs, are costly and take time to prepare.

The potential tumorigenicity of transplanted cells is a major issue of stem/progenitor cell therapy, especially when using iPSCs. Transplantation of insufficiently controlled iPSC products can lead to teratoma formation (Gutierrez-Aranda et al., 2010). Several quality control steps are essential to ensuring that such risks are minimized. The filtering of undifferentiated iPSCs or intermediate stem cell-like products should be carefully conducted, while avoiding somatic mutations due to prolonged culture, which can confer tumorigenic potential in the cells. These steps are necessary for each new cell line that is generated, making analogous therapies significantly a steeper challenge from patient sampling to bedside delivery. On the other hand, allogeneic cells may be expanded more efficiently and banked for application to a wider population. While the latter does require gene editing through ‘immune cloaking’ modifications, a few lines with sufficient B2M-knockout and HLA-E-knock-in may be generated and used across a larger population, compared to the one-to-one basis of analogous cell therapy (Cerneckis et al., 2024).

Integration and remodeling of regenerating muscle requires cooperation between several myotubes that have successfully differentiated by this phase in non-disease conditions. Without sufficient cell survival rates, fusion of newly differentiating myotubes is unlikely to occur with remaining dystrophic myotubes. Additionally, muscle fiber lifespan is linked to damage either physical, chemical or due to genetic mutation. In the context of DMD, by the time patients are diagnosed for potential treatments by age five to six, their muscles have already undergone multiple cycles of aberrant repair and fibrosis, limiting the number of myotubes available for normal fusion. Therefore, treatments must vitally look to improving the number of surviving grafted cells which can form new fibers by themselves. This may be combined by priming these cells before grafting towards differentiation, performing multiple transplants and using artificial scaffolds. These conditions will likely need to be adapted depending on the type of cell modality (MuSCs, myoblasts, DECs, etc.). It is not clear how long the transplanted cells will survive and function, or whether they can repopulate the depleted MuSC niche. However, if stem/progenitor cells were transplanted into dystrophic muscles by single or repeated intramuscular transplantation, clinical trials have shown insufficient muscle integration, likely because they may quickly die in a deteriorating environment of chronic inflammation, severe fibrosis, and fat infiltration. On the other hand, several clinical trials suggest that their paracrine effects may last longer than 12 months (Table 1). As such, early therapy for patients may include treatment of their muscles to accept stem/progenitor cells prior to grafting, either by minimizing fibrosis or inhibiting apoptotic conditions. Dystrophic muscles with a continuous degenerative state need repeated doses of stem/progenitor cells, which are costly and increase the risk of immune rejection. Therefore, it is crucial to generate a balance between over-stimulation of an immune response and maintaining conditions in the patient’s muscle to allow for grafted stem/progenitor cells to survive and differentiate. This will likely require strict patient-to-patient care and monitoring of short-term biomarkers for improvement between repeated treatments. This kind of therapy would require significant attention on the part of healthcare workers and therapy administrators.

**TABLE 1:** Clinical trials of stem/progenitor cell therapy for DMD.

Type of study	Dosages and route	Patients	Follow-up	Outcomes	References
Myoblasts					
Not Double-blind	4–155 × 10^6^ cells, Intramuscular, single or repeated doses (depending on site)	4	2 months	- Observed dystrophin-positive fibers in three patients (75%, 25%, and 80%, respectively) - Transiently improved muscle strength in one patient	Huard et al., (1992)
Double-blind	100 × 10^6^ cells, Intramuscular	8	1 month	- Restored normal dystrophin transcripts in three patients - Observed dystrophin-positive fibers (∼1%) in two patients	Gussoni et al. (1992)
Blind	55 × 10^6^ cells, Intramuscular	8	12 months	- Observed dystrophin-positive fibers (5%) and dystrophin immunoblot (5%) in one patient - Increased muscle voluntary contraction in three patients	Karpati et al. (1993)
Randomized, Double-blind	110 × 10^6^ cells, Intramuscular, monthly, 6 times	12	12 months	- Observed dystrophin-positive fibers (10.3%) in one patient	Mendell et al. (1995)
Double-blind	80–100 × 10^6^ cells, Intramuscular	20	6 months	- Restored dystrophin transcripts in three patients - Observed dystrophin-positive fibers (∼1%) in two patients	Miller et al. (1997)
Phase 1/2, Randomized	30 × 10^6^ cells, Intramuscular	10	6 months	- Not reported yet.	NCT02196467
Dystrophin-expressing chimeric cells (DT-DEC01)					
Non-randomized, Open-label	2 × 10^6^ cells/kg, Intraosseous	3	24 months	- Increased 6MWT in two patients - Improved NSAA scores in two patients - Increased grip strength in all patients - Improved electromyography in all patients - Improved or maintained EF and FS values in all patients - Improved FVC and FEV1 in one patient	Siemionow et al. (2023), Niezgoda et al. (2023)
(HOPE-2) Phase 2, Double-blind, Randomized	150 × 10^6^ cells, Intravenous, every 3 months, 4 times	20	12 months	- Improved PUL1.2 and 2.0 parameters - Improved cardiac MRI parameters, including LVEF, LVES volume, LVES volume, indexed, and LVED volume, indexed	McDonald et al. (2022) NCT03406780
(HOPE-2-OLE) Phase 2, Open-label extension of the HOPE-2	150 × 10^6^ cells, Intravenous, every 3 months, 20 times	13	60 months	- Ongoing	NCT04428476
(HOPE-3) Phase 3, Randomized, Double-blind, Placebo-controlled	150 × 10^6^ cells, Intravenous, every 3 months, 4 times	104	12 months	- Ongoing	NCT05126758
Mesoangioblasts					
Phase 1/2, Non-randomized, Open-label	3.3–19.8 × 10^6^ cells, Intraarterial, every 2 months, 4 times	5	2 months	- Improved NSAA score and 6MWT in one patient - Sustained time to run 10 m in one patient - Detected dystrophin protein expression in one patient	Cossu et al. (2015) Eudract 2011–000176–33
CD133^+^ cells					
Phase 1, Double-blind	2 × 10^4^ cells, Intramuscular	8	7 months	- Improved maximal isometric voluntary contraction (MVC) of left abductor digiti minimi in two patients - Increased number of capillaries per myofiber	Torrente et al. (2007)
iPSC-derived CD54^+^ myogenic progenitors					
Phase 1, Non-randomized, Open-label	25, 50, 100, and 200 × 10^6^ cells/patient	8	3 months	- Recruiting	NCT06692426

To improve the efficacy of cell-based therapies, it is first necessary to understand the traits of successful therapies that allowed them to graft, while minimizing the aspects that prevent them from doing so. It is crucial to comprehend why promising therapies at the pre-clinical stage frequently have diminished effects during early or mid-phase clinical trials. From our current understanding, there is no clear outlier cell type, route of administration or pre-treatment that has led to a marked improvement of both stable expression of dystrophin in DMD tissue or long-term survival and replenishment of the MuSC pool. However, we may navigate trends to consider a path forward for future strategies. Additionally, understanding why low survival rates of grafted cells may be crucial to generating more consistent cell-based therapies. For example, performing transplantation *in vivo*, followed by short-term recovery to perform high-throughput single-cell RNA sequencing to identify how transplanted cells were modified by the disease niche, the percentage of cells that differentiated towards regenerative pathways and the trajectories of those that did not. As many strategies test the engraftment of healthy or corrected human cells into a murine system, separation of the signals would be technically straightforward. Results from such studies would reveal the weaknesses of current therapies and potentially allow us to design more reliable modalities.

Economic considerations are a crucial factor for patients, healthcare providers and governments seeking to adapt these therapies. For a male patient weighing 30 kg, a weekly intravenous administration of viltolarsen (80 mg/kg) is expected to cost approximately \47,000,000 per year in Japan and $700,000 in the US. A single administration of ELEVIDYS is expected to cost approximately 3,200,000 (equivalent to \470,000,000 at the time of publication, for reference only due to differences in the drug pricing system between Japan and the US) in the US. In the case of MSC therapies for other diseases, BM-MSCs (STEMIRAC developed by NIPRO) for spinal cord injury is approximately \15,000,000 (equivalent to 100,000) per use, BM-MSCs (TEMCELL developed by JCR Pharmaceuticals) for steroid-refractory acute graft *versus* host disease is approximately \8,700,000 (equivalent to 59,000). These MSC therapies are intended for acute disease and are more costly for DMD treatment due to the requirement of repeated dosing. On the other hand, robotics in stem/progenitor cell therapies have been progressing in recent years (Kanda et al., 2022), and such automation technologies are expected to significantly lower costs of stem/progenitor cell therapy development in the future.

## 6 Conclusion

Stem/progenitor cell transplantation holds significant promise for the treatment of DMD. In Figure 4B we have illustrated the targeting of several stem/progenitor cell modalities into dystrophic muscle as potential therapies. The field of stem/progenitor cell transplantation for DMD is rapidly evolving, with several emerging technologies and strategies. The last few years have seen significant strides in advancing stem/progenitor cell therapies for improving the regeneration of dystrophic muscle and restoring dystrophin protein in DMD patients. Wide-ranging strategies to target the numerous aspects of DMD pathology have been published, particularly improving the survival of grafted healthy or corrected MuSCs and increasing their longevity in the disease niche. In particular, the improved ambulation observed in patients grafted with DECs represents an important step towards using allogeneic cells in future studies. Many of these trials were limited to small cohorts and relatively short follow-up times relative to disease progression. Thus, future long-term studies with more participants will be crucial to establishing such cell-based therapies as viable treatments for patients. Additionally, with a significant number of pre-treatments that have been successful in pre-clinical studies, the scope for combinatorial therapies is also widening. Finally, the emergence of CDCs and CDC-exosomes as a potential for targeting the main cause of death in DMD is a particular point of optimism for the future of therapy in this field. While several challenges remain relating to matching healthy donors to patients and identifying the correct regimen, the numerous advancements give us assurance for the future.

Consistent with almost all allogeneic stem/progenitor cell-based therapies, there are discrepancies between pre-clinical studies and clinical studies. The engraftment of transplanted stem/progenitor cells into dystrophic muscles, dystrophin restoration, and functional improvement via intramuscular or intravenous injection is often robust in DMD animal models, but less so in humans. These discrepancies may be due to several issues including immune rejection against allogeneic cell transplantation and cell delivery, or the condition of the human muscle compared to animal models (Maffioletti et al., 2014; Saleh et al., 2023). Immune rejection against transplanted cells and/or newly expressed dystrophin protein is the primary reason limiting the efficacy of cell transplantation. This issue is also observed in other therapeutic approaches that use viral vector-mediated gene therapy in the treatment of DMD, resulting in discrepancies in therapeutic efficacy between animal models and humans. Immunosuppressants, such as tacrolimus, have been used to mitigate this issue in humans; however, they do not completely suppress the immune rejection and have adverse effects, such as the risk of infection and organ dysfunction. Although the use of MSCs can be expected to modulate immune responses, further validation is needed as it has been reported that the alloreactive response of MSCs led to donor cell rejection (Nauta et al., 2006). Intramuscular transplantation of high-density allogeneic stem/progenitor cells can restore dystrophin protein expression (Skuk et al., 2006), but local transplantation has limited therapeutic efficacy in systemic muscle pathology of DMD, and there is an urgent need to develop a cell-delivery method that can be targeted efficiently to dystrophic muscles via systemic administration. Otherwise, gene-edited or iPSC-derived cells can be autologously grafted to reduce the risk of immune rejection, but preparing them for transplantation is complex and costly. While there is a clear potential for various pretreatments of myogenic cells and stem cells to improve their engraftment, their application in a clinical setting is yet to be explored and requires significant dosage and safety investigations. In addition, examination in a DMD paradigm is yet lacking in several mouse studies, that instead used induced-injury or ageing models.

## 7 Future perspectives

Stem/progenitor cell therapy was initially developed to replenish dystrophin-expressing myofibers or the MuSC pools. However, systemic delivery to skeletal and cardiac muscles throughout the body remains highly challenging. Consequently, current research has shifted its focus to the paracrine effects of transplanted cells. In fact, most stem/progenitor cells currently undergoing clinical trials for DMD exert their therapeutic effects primarily through paracrine effects (Tables 1, 2).

**TABLE 2:** Advantages and disadvantages of stem/progenitor cell therapies.

Type of study	Mechanism of action	Advantages (including potential)	Limitations	Clinical trials
Myoblasts (allogeneic)	- Promoting muscle regeneration - Dystrophin restoration	- Restoration of dystrophin expression - Functional improvement of skeletal muscle	- Loss of potency and difficulty in scaling up *ex vivo* expansion - Poor cell engraftment and survival - Not suitable for systemic transplantation due to limited cell migration (stays at the injection site) - Little restoration of dystrophin expression due to inefficient fusion - Difficulty in targeting the diaphragm and heart - Limited effects due to dystrophic muscle associated with fat and fibrotic infiltrations in severe fibrosis in progressed DMD. - Immune rejection	Yes
Muscle stem cells (allogeneic)	- Promoting muscle regeneration - Dystrophin restoration - Replenishing the stem cell pool	- Replenishment of the stem cell pool - Restoration of dystrophin expression - Functional improvement of skeletal muscle	- Limited the number of MuSCs isolated from muscle biopsies - Loss of stemness and potency in scaling up *ex vivo* expansion - Poor cell engraftment and survival - Not suitable for systemic transplantation due to limited cell migration (stays at the injection site) - Difficulty in targeting the diaphragm and heart - Exhaustion of transplanted cells due to the rapid cycle of injury-regeneration in dystrophic muscle - Immune rejection	No
iPSC-derived myogenic progenitors (autologous)	- Promoting muscle regeneration - Dystrophin restoration	- Autologous transplantation - Unlimited cell source - Patient-specific model - Potential for the restoration of dystrophin expression via fusion with host muscle cells - Improvement of skeletal muscle function	- Tumorigenicity risk - Need for protocol optimization for myogenic differentiation - Poor cell engraftment and survival - Not suitable for systemic transplantation due to limited cell migration (stays at the injection site) - Difficulty in targeting the diaphragm and heart - Off-target effects by gene editing - Complexity of the manufacturing process and high costs	No
Dystrophin-expressing chimeric cells (Mixed autologous/allogeneic)	- Promoting muscle regeneration - Dystrophin restoration	- Potential for the restoration of dystrophin expression - Transfer of healthy mitochondria - Improvement of skeletal muscle function - Systemic intraosseous transplantation - Lower risk of immune rejection due to chimeric cells from the healthy control and the patient	- Dystrophin restoration in humans has not yet been proven - Poor cell engraftment and survival - Need for the optimization of the cell-to-cell fusion protocol - Insufficient number of patients in clinical trials	Yes
Mesenchymal stem cells (allogeneic)	- Promoting muscle regeneration by paracrine effects	- Easy to isolate from several tissues, such as bone marrow, fatty tissue and umbilical cord - Relatively scalable compared to other cell types - Dystrophin restoration (theoretically, but unrealistic) - Paracrine effects - Low immunogenicity - Functional improvement of skeletal muscle - Systemic delivery - Possibility of combining therapies with other approaches	- Poor cell engraftment and survival - Unreliable myogenic differentiation and dystrophin restoration - Limited functional improvement of skeletal muscle - Heterogeneity - Lack of standardization	Yes
Cardiosphere-derived cells (allogeneic)	- Promoting muscle regeneration by cell engraftment and/or paracrine effects	- Functional improvement of both cardiac and skeletal muscles - Exosome-mediated effects (paracrine effects) - Systemic delivery - A favorable safety profile	- Transient effect on dystrophin restoration - Poor cell engraftment and survival - Immune rejection	Yes
Mesoangioblasts (allogeneic)	- Promoting muscle regeneration	- Crossing blood vessel walls - Systemic delivery - Dystrophin restoration - Paracrine effects - Functional improvement of skeletal muscles - A favorable safety profile	- Limited functional improvement of skeletal muscles - Poor cell engraftment and survival - Side effect (Livedo reticularis in two patients and acute small thalamic stroke in one patient) - Immune rejection	Yes
CD133+ cells (autologous)	- Angiogenesis - Promoting muscle regeneration by paracrine effects	- Pro-angiogenesis - Paracrine effects - Dystrophin restoration - Functional improvement of skeletal muscles - Lower risk of immune rejection - A favorable safety profile	- Limited functional improvement of skeletal muscles - Poor cell engraftment and survival	Yes
iPSC-derived CD54^+^ myogenic progenitors (allogeneic)	- Promoting muscle regeneration - Dystrophin restoration - Replenishing the stem cell pool	- Dystrophin restoration - Functional improvement of skeletal muscles - Replenishment of the stem cell pool	- Under consideration	Planned

Currently, we are conducting studies focusing on urine-derived stem cells (UDCs) and multilineage-differentiating stress-enduring (Muse) cells as novel candidates for stem/progenitor cell-based therapy. UDCs, an MSC-like cell type, can be collected non-invasively and repeatedly from donors or patients, and have higher proliferative properties compared to other stem/progenitor cells, including BM-MSCs, and multipotency (Zhang D. et al., 2014). In addition, UDCs can be a potential compatibility for autologous transplantation by gene correction. Our previous studies demonstrated that CD90/Thy-1-positive UDCs possess high myogenic potential upon transduction with MyoD1 (Kunitake et al., 2024). Although UDCs offer significant advantages, optimization and standardization of the protocol remain essential to control cell heterogeneity and cell quality depending on the timing of their collection. They represent a promising alternative for personalized cell therapies in DMD. On the other hand, Muse cells have been identified as a rare subpopulation of MSCs, which possess pluripotency. They can spontaneously migrate to injured muscle and differentiate into PAX7-positive cells in wild-type mice (Kuroda et al., 2010). Additionally, they have been reported to phagocytose differentiated cell fragments and differentiate into corresponding cell types by utilizing tissue-specific transcription factors derived from the engulfed cells (Wakao et al., 2022). These stem cell types hold promise as a new source of stem/progenitor cell-based therapy for DMD.

As stem/progenitor cell therapies are intended to ameliorate dystrophic pathologies by paracrine effects, by improving the dystrophic microenvironment. Thus, the combined strategies with other approaches can be effective for treating DMD. Cardiomyopathy is a leading cause of death in patients with DMD. Recently, engineered heart muscle implantation created by iPSC-derived cardiomyocytes and stromal cells showed significant efficacy for heart failure after myocardial infarction in animal models and humans (Jebran et al., 2025). Another group demonstrated that UDCs successfully differentiate into functional cardiomyocytes, and that transplantation of UDC-cardiomyocytes into a swine myocardial infarction model improves cardiac function (Chen Y. et al., 2025). Considering these findings, an engineered heart patch composed of UDCs or MSCs embedded in scaffolds such as hydrogel may represent an effective approach for cardiomyopathy in DMD. Furthermore, a differentiated treatment strategy for cardiac and skeletal muscles could be effective, utilizing a heart patch engineered from UDCs or MSCs for treating cardiomyopathy and applying exon-skipping therapy, which has limited cardiac delivery, for treating skeletal muscle pathologies.

DMD models generated by our group, including CXMD_J_ dogs (Shimatsu et al., 2003) and DMD-edited microminipigs (Otake et al., 2024), may be more effective to investigate stem/progenitor cell therapeutic efficacy as they exhibit muscle pathology more closely resembling humans. Future studies should focus on optimizing delivery routes, exploring combinations with other therapeutic approaches, elucidating the utilization of biomaterials, and developing scalable and cost-effective protocols for cell preparation.

## Footnotes

1 Sarepta Therapeutics. Sarepta Provides Safety Update for ELEVIDYS and Initiates Steps to Strengthen Safety in Non-Ambulatory Individuals with Duchenne. https://investorrelations.sarepta.com/news-releases/news-release-details/sarepta-provides-safety-update-elevidys-and-initiates-steps. Accessed on 24 July 2025.

2 Sarepta Therapeutics. Sarepta Therapeutics Announces Voluntary Pause of ELEVIDYS Shipments in the U.S. https://investorrelations.sarepta.com/news-releases/news-release-details/sarepta-therapeutics-announces-voluntary-pause-elevidys. Accessed on 24 July 2025.
